# RacGAP1 Plays an Oncogenic Role in Lung Adenocarcinoma by Regulating the Wnt/β-Catenin Pathway

**DOI:** 10.3390/cells14110773

**Published:** 2025-05-23

**Authors:** Nicola Mosca, Mariaceleste Pezzullo, Ilenia De Leo, Anna Truda, Giovanna Marchese, Aniello Russo, Nicoletta Potenza

**Affiliations:** 1 Department of Environmental, Biological and Pharmaceutical Sciences and Technologies, University of Campania “Luigi Vanvitelli”, 81100 Caserta, Italy; nicola.mosca@unicampania.it (N.M.); mariaceleste.pezzullo@unicampania.it (M.P.); ilenia.deleo@unicampania.it (I.D.L.); anna.truda@unicampania.it (A.T.); aniello.russo@unicampania.it (A.R.); 2 Genomix 4Life S.r.l., 84081 Baronissi, Italy; giovanna.marchese@genomix4life.com; 3 Genome Research Center for Health-CRGS, 84081 Baronissi, Italy

**Keywords:** lung adenocarcinoma, GAP, GTPase activating protein, siRNA silencing, Wnt pathway

## Abstract

Lung cancer is the most diagnosed cancer and the primary cause of cancer-related mortality worldwide, with lung adenocarcinoma (LUAD) becoming the prevalent histological subtype. Rac GTPase activating protein 1 (RacGAP1) has been found to be upregulated in several cancers, where it acts as an oncogene; nevertheless, its role in lung adenocarcinoma is largely unknown. The present study investigated the clinical relevance, the oncogenic function and the underlying molecular mechanisms of RacGAP1 in LUAD. Analyses of five patient cohorts’ datasets revealed that RacGAP1 was upregulated in adenocarcinoma tissues compared to normal lung tissues, and its overexpression was associated with unfavorable prognostic factors and poor survival; intriguingly, RacGAP1 expression was related to tobacco smoke, a well-known risk factor for LUAD. Then, experimental analyses demonstrated that RacGAP1 knockdown inhibited cell proliferation, migration and invasion, thus highlighting its role in promoting LUAD. Finally, the finding of significant correlations between RacGAP1 and Wnt-altered status or β-catenin in patients led to experiments demonstrating that silencing of RacGAP1 reduced β-catenin transcriptional activity, thereby downregulating the expression of Wnt-related genes, i.e., LGR5, Wnt2B and Wnt5A. Overall, our findings indicate that RacGAP1 plays an oncogenic role in adenocarcinoma, contributing to the abnormal activation of the Wnt/β-catenin signaling pathway. These findings may pave the way for innovative therapeutic strategies and the development of advanced diagnostic panels.

## 1. Introduction

Lung cancer (LC) is the most diagnosed cancer, with 2.5 million new cases and 1.8 million new deaths reported worldwide in 2022. It accounts for 12.4% of all cancer diagnoses and 18.7% of all cancer-related deaths. LC has the highest incidence and mortality rates among men and ranks second in both incidence and mortality among women [1,2].

LC is categorized into two main histological types: small cell lung cancer (SCLC) and non-small cell lung cancer (NSCLC). SCLC represents approximately 15–20% of cases and is strongly associated with cigarette smoking [3]. NSCLC, accounting for approximately 85% of cases, is further subclassified into different subtypes, including lung adenocarcinoma (LUAD), lung squamous cell carcinoma (LUSC), large cell carcinoma and bronchial carcinoid tumor, with LUAD emerging as the most prevalent subtype within NSCLC [4]. In addition to chemotherapy based on histopathology, the identification of predictive biomarkers and oncogenic-driven mutations (such as EGFR, ALK, ROS1, BRAF, RET, MET and HER2) has led to the development of targeted therapy and immunotherapy [4,5,6]. Interestingly, many of these oncogenes are key players in pathways regulating cell growth, and their mutations lead to cause alterations in the cell cycle driving cells through the restriction point in G1 and culminating in the activation of the G1–S transcriptional program [7].

Rho GTPases, a subgroup of the Ras superfamily, are important mediators of actin and microtubule cytoskeleton dynamics and act as molecular switches on the basis of their GTP- or GDP-bound state. They can regulate cell cycle entry and progression, including the G1/S transition, and are critical for mitosis [8]. Rho GTPase-regulating proteins, including GTPase-activating proteins (Rho GAPs) that inactivate Rho GTPases by promoting GTP hydrolysis, often show altered expression or mutations in cancers [9].

Rac GTPase-activating protein 1 (RacGAP1), also known as MgcRacGAP, is a member of the Rho GAPs that exhibits GAP activity toward Rac and Cdc42 [10] and is involved in various cellular processes, including cytokinesis and assembly of the contractile ring [11]. Studies have shown that RacGAP1 promotes cell proliferation, migration and invasion as well as its overexpression is associated with various cancers [9,11,12]. In hepatocellular carcinoma (HCC), RacGAP1 supports tumor growth by suppressing the Hippo/YAP pathway [13]. In cervical cancer, it activates the PI3K/AKT pathway, increasing cell viability and invasiveness [14]. High RacGAP1 levels are correlated with poor prognosis in esophageal cancer, where RacGAP1 knockdown reduces proliferation and metastasis [15]. Similar oncogenic roles are observed in glioma and ovarian cancer [16,17]. Moreover, recent findings suggest the potential involvement of RacGAP1 in the regulation of the Wnt pathway in colorectal cancer and gastric cancer [18,19]. The Wnt signaling pathway plays a critical role in many cancers, including LC [20], contributing to promoting proliferation, migration and invasion [21,22], inhibiting apoptosis [22,23], maintaining stemness [24], driving the EMT process [25], promoting angiogenesis [26] and facilitating metastasis [25,26,27]. With regard to LC, very recently, RacGAP1 has been found to be upregulated in tumor samples and associated with the PI3K/AKT signaling pathway in the regulation of cell proliferation [28,29,30]; however, the clinical significance of RacGAP1 overexpression and mechanistic insights on how it is involved in pathways driving lung cancer are still largely unknown.

The present study aimed to investigate the clinical relevance, potential oncogenic role and molecular mechanisms of RacGAP1 in LUAD. After demonstrating dysregulation of RacGAP1 in LUAD at both the transcript and protein levels, as well as its associations with clinical parameters, the oncogenic role of RacGAP1 was experimentally assessed by using cell-based assays. Finally, its association with Wnt/β-catenin signaling was demonstrated in LUAD by in silico and experimental analyses.

## 2. Materials and Methods

### 2.1. Bioinformatics Analysis

The transcriptomic data were analyzed by R2 (Genomics analysis and visualization platform, https://r2.amc.nl accessed on 10 October 2023). The datasets TCGA-LUAD, GSE31210, GSE10072, GSE33532 and GSE19188 were downloaded from R2, and RacGAP1 expression was analyzed for correlation with clinical features and survival. The proteomic data were analyzed via the UALCAN (The University of ALabama at Birmingham CANcer data analysis Portal, https://ualcan.path.uab.edu/index.html accessed on 10 October 2023) database.

### 2.2. Cell Culture and Transfection

The A549 human lung adenocarcinoma cell line (ATCC, Manassas, VA, USA) was cultured in DMEM containing 10% fetal bovine serum, 50 U/mL penicillin and 100 µg/mL streptomycin. The Calu-3 human lung adenocarcinoma cell line (ATCC, Manassas, VA, USA) was cultured in MEM supplemented with 1% non-essential amino acid (NEAA), 1 mM of sodium pyruvate, 10% fetal bovine serum, 50 U/mL penicillin and 100 µg/mL streptomycin. Cells were tested monthly for mycoplasma-free infection. The cells were trypsinized and seeded in medium without antibiotics the day before transfection. At 80–90% confluence, the cells were transfected with 50 nM of Mission^®^ esiRNA RacGAP1 (EHU081301, Merck, Darmstadt, Germany), a heterogeneous pool of siRNAs effectively targeting the transcript, or Mission siRNA Universal Negative Control #1 (SIC001, Merck, Darmstadt, Germany), using 2 µL Lipofectamine 2000 (Invitrogen, Thermo Fisher Scientific, Waltham, MA, USA) per 1 µg of nucleic acids. Six hours post-transfection, the transfection mix was replaced with complete growth medium. Analyses were performed 48 h after transfection.

### 2.3. Cell Proliferation

The MTT assay was used to evaluate cell proliferation. Briefly, cells were plated in 96-well plates, and transfection was performed as described above. After 48 h, cell growth was evaluated by adding 150 µL of complete medium containing 0.5 mg/mL 3-(4,5-dimethylthiazol-2-yl)-2,5-diphenyltetrazolium bromide (MTT) to each well. Following a 1 h incubation at 37 °C, the medium was removed and the formazan crystals produced in viable cells were solubilized in 100 µL of dimethyl sulfoxide and quantified by measuring the absorbance at 570 nm with the GloMAX discover system (Promega, Madison, WI, USA).

### 2.4. Cell Migration

A wound-healing assay was used to evaluate cell migration. Briefly, cells were transfected into a 12-well plate as described above. A uniform scratch was made on the plate using a sterile 200-μL pipette tip when the cells reached confluence. Detached cells from scratch were removed by PBS washing, followed by the addition of fresh serum-free culture medium. Empty space colonization by cells was imaged using microscope at 0, 24 and 48 h. Quantification was performed by Image J 1.52a.

### 2.5. Cell Invasion

Transwell assay was used to evaluate cell invasion. Briefly, A549 cells were transfected into 12-well plates as described above. After 24 h, the cells were trypsinized and plated in serum-free medium in the upper chamber of the transwell, which was precoated with Geltrex (Gibco, Thermo Fisher Scientific, Waltham, MA, USA). The lower chamber was filled with medium containing 10% FBS. After 16 h of incubation, the invading cells were fixed with 4% PFA and subsequently stained by crystal violet. Five random fields were imaged by the microscope. Quantification was performed using Image J 1.52a.

### 2.6. Top Flash Assay

A549 cells were seeded in a volume of 100 µL in 96-well plates. Then, 0.025 µg pRL-TK-Renilla (Promega, Madison, WI, USA) and 0.065 µg pGL4-TOP plasmids [31,32] were co-transfected with 50nM RacGAP1 siRNA (or siRNA control) using Lipofectamine 2000 (Invitrogen, Thermo Fisher Scientific, Waltham, MA, USA). After 48 h, the cells were lysed, and luciferase activity was measured using the Dual-Luciferase Reporter Assay System (Promega, Madison, WI, USA) according to the manufacturer’s instructions by GloMAX discover system (Promega, Madison, WI, USA).

### 2.7. RNA Purification and Real-Time PCR Analysis

The miRNeasy mini kit (Qiagen, Hiden, Germany) was used to isolate total RNA from cell cultures according to the manufacturer’s instructions. Total RNA was retrotranscribed using SensiFAST cDNA Synthesis Kit (Bioline, London, UK). Standard SYBR Green real-time PCR assays were then performed to quantify messenger RNA (mRNA) expression. The PCR primers are listed below:

RacGAP1, F: 5′-GAAAGCAGAGACTGAGCGAAG-3′ and R: 5′-GTTGAATGCTGCCAGATGTGT-3′;

β-catenin, F: 5′- TCTTACACCCACCATCCCAC-3′ and R: 5′-GCACGAACAAGCAACTGAAC-3′;

CCND1, F: 5′-TGGCGTTTCCCAGAGTCATC-3′ and R: 5′-AAGGAAGGGGCAGGGGATAA-3′;

Wnt2B, F: 5′-ATTTCCCGCTCTGGAGATTT-3′ and R: 5′-GGTACCCTTCCTCTTGCACA-3′;

Wnt5A, F: 5′-CAAGGGCTCCTACGAGAGTG-3′ and R: 5′-CCCACCTTGCGGAAGTCT-3;

LGR5, F: 5′- GGAGTTACGTCTTGCGGGAA-3′ and R: 5′-TGGTTAGCATCCAGACGCAG-3′;

GAPDH (reference transcript), F: 5′-GAAGGTGAAGGTCGGAGTC-3′ and R: 5′-GAAGATGGTGATGGGATTT-3′.

GAPDH mRNA was used as an internal control for normalization. The 2^−ΔΔCt^ method [33] was used to normalize the transcript expression levels to those of the control condition.

### 2.8. Nuclear and Cytoplasmic Protein Extraction

Protein samples were extracted from nuclear and cytoplasmic fractions. Briefly, the cells were resuspended in 1 mL of PBS and centrifuged at 500× *g* for 5 min (4 °C). The cell pellet was resuspended in 50 µL of solution A (10 mM Hepes pH 7.9, 1.5 mM MgCl2, 10 mM KCl, 1 mM Na3VO4, 1 mM PMSF, 1 mg/mL Leupeptin), incubated on ice for 15 min, mixed by vortexing and then centrifuged at 10,000× *g* for 15 min (4 °C). The supernatant containing the cytoplasmic protein fraction was collected. The pellet was washed twice with solution A and then resuspended in 50 µL of solution B (20 mM Hepes pH 7.9, 25% glycerol, 420 nm NaCl, 1.5 mM MgCl2, 0.2 mM EDTA pH8.0, 1 mM Na3VO4, 1 mM PMSF, 1 mg/mL Leupeptin), incubated for 20 min on ice, mixed by vortexing and then centrifuged at 10,000× *g* for 15 min (4 °C). The supernatant containing the nuclear protein fraction was collected. Protein concentrations were determined using the Pierce BCA Protein Assay Kit (Thermo Scientific, Waltham, MA, USA) according to manufacturer’s instructions.

### 2.9. Western Blotting

A total of 30 μg of proteins were separated on a denaturing 12% polyacrylamide gel and then blotted onto nitrocellulose membranes (0.2 µm pore size, Thermo Scientific, Waltham, MA, USA). The membranes were blocked in 5% skim milk and then incubated with the following specific primary antibodies: Anti-RacGAP1 (1:1000, #ab97315, Abcam, Cambridge, UK), anti-β-catenin (1:3000, #8480, Cell Signaling Technology, Danvers, MA, USA), anti-Histone H3 (1:2000, #4499, Cell Signaling Technology, Danvers, MA, USA), anti-GAPDH (1:5000, #sc-25778, Santa Cruz Biotechnology, Dallas, TX, USA), anti-α-Tubulin (1:1000, LF-PA0146, Abfrontier, Seoul, Republic of Korea) at 4 °C overnight. After washing three times with 0.05% Tween-TBS, the membranes were incubated with secondary Goat Anti-Rabbit IgG (HRP) antibody (1:5000, ab97051, Abcam, Cambridge, UK). The immunoreactions were detected using WesternBright ECL HRP substrate (Advansta, San Jose, CA, USA) and the Chemi Doc XRS+ (Bio-Rad, Hercules, CA, USA).

### 2.10. Statistics

All the data are presented as the means of three or more independent experiments. The error bars indicate the standard deviation (SD) of the mean. Statistical analyses were performed using GraphPad Prism 9.1 software. Comparisons of datasets across different experiments were performed using Student’s *t*-test or the nonparametric Mann-Whitney test for experiments involving two groups of values. When the experiment included three groups of values or more, one-way analysis of variance (ANOVA) was used for the comparison of multiple means. When experiments included two sets of categorical variables (e.g., <median versus ≥median), the two-tailed chi-square test was used. The Shapiro–Wilk test was used to assess the normality assumptions. The results with a *p*-value < 0.05 were considered statistically significant.

## 3. Results

### 3.1. RacGAP1 in Patients

**Figure 1:**
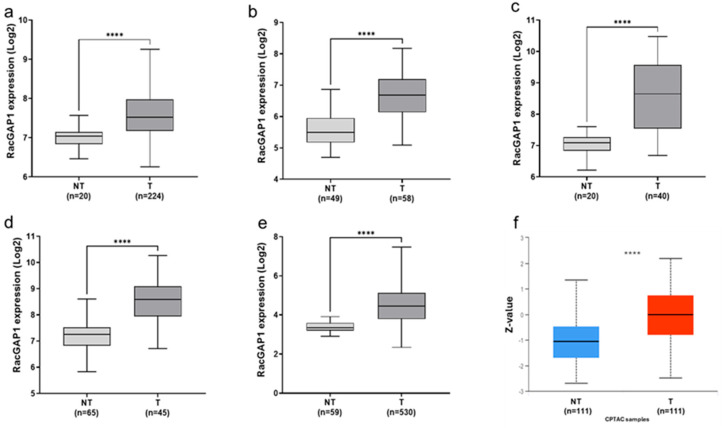
RacGAP1 expression in lung adenocarcinoma tissues and normal tissues. RacGAP1 mRNA levels in lung adenocarcinoma (T) and non-tumoral (NT) samples from (**a**) GSE31210, (**b**) GSE10072, (**c**) GSE33532, (**d**) GSE19188 and (**e**) TCGA-LUAD datasets from the R2 platform (https://r2.amc.nl accessed on 10 October 2023). (**f**) Box plot reporting RacGAP1 protein levels (Z-value) in tumoral (T) and non-tumoral (NT) samples from the Clinical Proteomic Tumor Analysis Consortium (CPTAC) dataset using the UALCAN database (https://ualcan.path.uab.edu/index.html accessed on 10 October 2023). **** *p* < 0.0001 for Mann-Whitney test (**a**,**b**,**e**,**f**) and Unpaired *t*-test (**c**,**d**).

The relevance of RacGAP1 in LUAD was evaluated by analyzing five different transcriptomic datasets of LUAD patients using the R2 platform. The results demonstrated that the RacGAP1 transcript was markedly upregulated in tumoral tissues compared with normal lung tissues in all datasets, with fold changes of 1.8 (GSE31210; *p* < 0.0001) (Figure 1a), 2.4 (GSE10072; *p* < 0.0001) (Figure 1b), 3.8 (GSE33532; *p* < 0.0001) (Figure 1c), 2.7 (GSE19188 *p* < 0.0001) (Figure 1d) and 2.7 (TCGA-LUAD; *p* < 0.0001) (Figure 1e). Additionally, the protein levels of RacGAP1 in LUAD patients were evaluated through the analysis of Clinical Proteomic Tumor Analysis Consortium (CPTAC) samples using the University of ALabama at Birmingham CANcer Data Analysis Portal (UALCAN). The data indicate that RacGAP1 protein levels were significantly higher in LUAD tissues than in normal lung tissues (Figure 1f). Taken together, these findings demonstrate that RacGAP1 was upregulated in LUAD at both the mRNA and protein levels.

**Figure 2:**
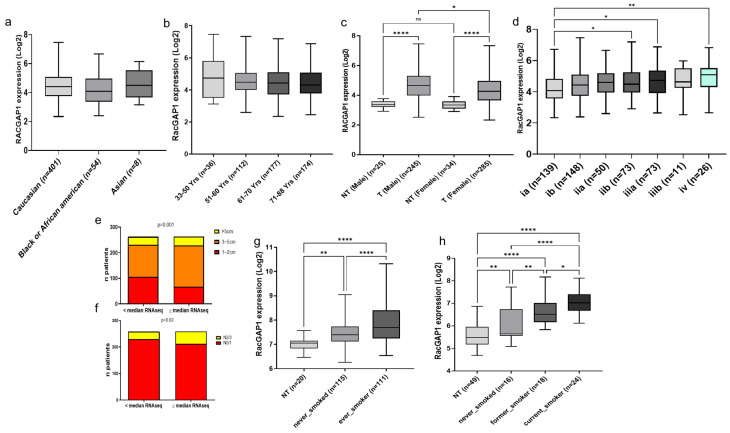
Correlation between RacGAP1 expression and clinical features in lung adenocarcinoma patients. Box plots showing RacGAP1 expression related to (**a**) race, (**b**) age, (**c**) sex, (**d**) tumor stage from TCGA dataset. Stacked bar graphs showing RacGAP1 expression related to (**e**) tumor size or (**f**) lymph node metastasis, referred to the number of patients (Y axis) in the two groups characterized by RacGAP1 expression < median RNAseq and ≥ median RNAseq (X axis); analyses were performed on LUAD patients from TCGA dataset by chi-square test. RacGAP1 expression in non-tumoral (NT) samples and tumoral sample from patients with different smoking habits in (**g**) GSE31210 and (**h**) GSE10072 datasets from the R2 platform. The number of patients is reported for each group (*n* =). ns: not significant, * *p* < 0.05, ** *p* < 0.01, **** *p* < 0.0001 for one-way ANOVA (**a**–**d**,**g**,**h**) and chi-square test (**e**,**f**).

Furthermore, the analysis of all datasets was performed to obtain information regarding RacGAP1 expression related to race, age, sex, tumor size, disease stage, nodal metastasis, smoking habits and survival, wherever reported, thus allowing the evaluation of the relationships between RacGAP1 expression and clinicopathological features in patients with lung adenocarcinoma. The TCGA-LUAD dataset revealed no differences in RacGAP1 expression levels among tumoral samples from Caucasian, Black/African American and Asian patients (Figure 2a), and no differences were found related to different ages (Figure 2b). In contrast, RacGAP1 was significantly upregulated in both male and female patients compared with non-tumoral samples, with higher levels observed in male patients than in female patients (Figure 2c). Moreover, RacGAP1 expression increased along the tumor stage, particularly in stage IV, which is the most advanced stage with metastasis or cancer spread, to the lining of the lung or other areas of the body (Figure 2d). Importantly, RacGAP1 expression was also correlated with several unfavorable prognostic factors, including tumor size (*p* = 0.001) (Figure 2e) and lymph node metastasis (*p* = 0.02) (Figure 2f). We also analyzed the relationship between RacGAP1 expression and common lung adenocarcinoma driver gene state (mutated and non-mutated), in particular EGFR, ALK and K-RAS, finding no obvious and significant difference. The expression of RacGAP1 was also analyzed in patients with different cigarette smoking histories. Interestingly, the expression of RacGAP1 was higher in smokers than in nonsmokers in two different datasets, GSE31210 and GSE10072, with fold changes of 1.4 (*p* < 0.0001) and 1.9 (*p* < 0.0001), respectively (Figure 2g,h). Subsequently, a comparison between smokers and former smokers revealed that the expression level of RacGAP1 was lower in patients who stopped smoking than in smokers but was higher than that in never smokers (Figure 2h).

**Figure 3:**
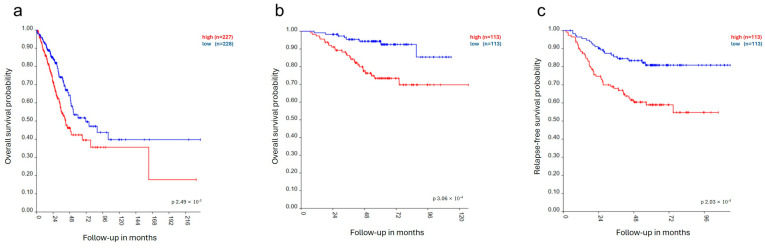
Correlation between RacGAP1 expression and survival in lung adenocarcinoma patients. The correlation between RacGAP1 expression (red line: high expression; blue line: low expression according to median value) and overall survival (reported as follow-up months) was analyzed through the Kaplan–Meier plotter on (**a**) TCGA-LUAD and (**b**) GSE31210 datasets using the R2 platform. (**c**) Correlation analysis between RacGAP1 expression and relapse-free survival performed on GSE31210 dataset. *p*-values were calculated by two-tailed chi-square test and are reported on the graphs.

Finally, the correlation between RacGAP1 expression and overall survival in LUAD patients was evaluated by analyzing the TCGA-LUAD and GSE31210 datasets. The analyses revealed that RacGAP1 expression was significantly correlated with decreased overall survival in both datasets, with *p*-values of 0.0003 (Figure 3a) and 0.002 (Figure 3b), respectively. RacGAP1 expression was also significantly correlated with decreased relapse-free survival (*p* = 0.0002) in the GSE31210 dataset (Figure 3c). Overall, these data suggest that RacGAP1 may function as an oncogene in lung adenocarcinoma and that its expression is associated with an unfavorable prognosis and poor survival.

### 3.2. Effects of RacGAP1 Silencing on Cell Proliferation, Migration and Invasion

**Figure 4:**
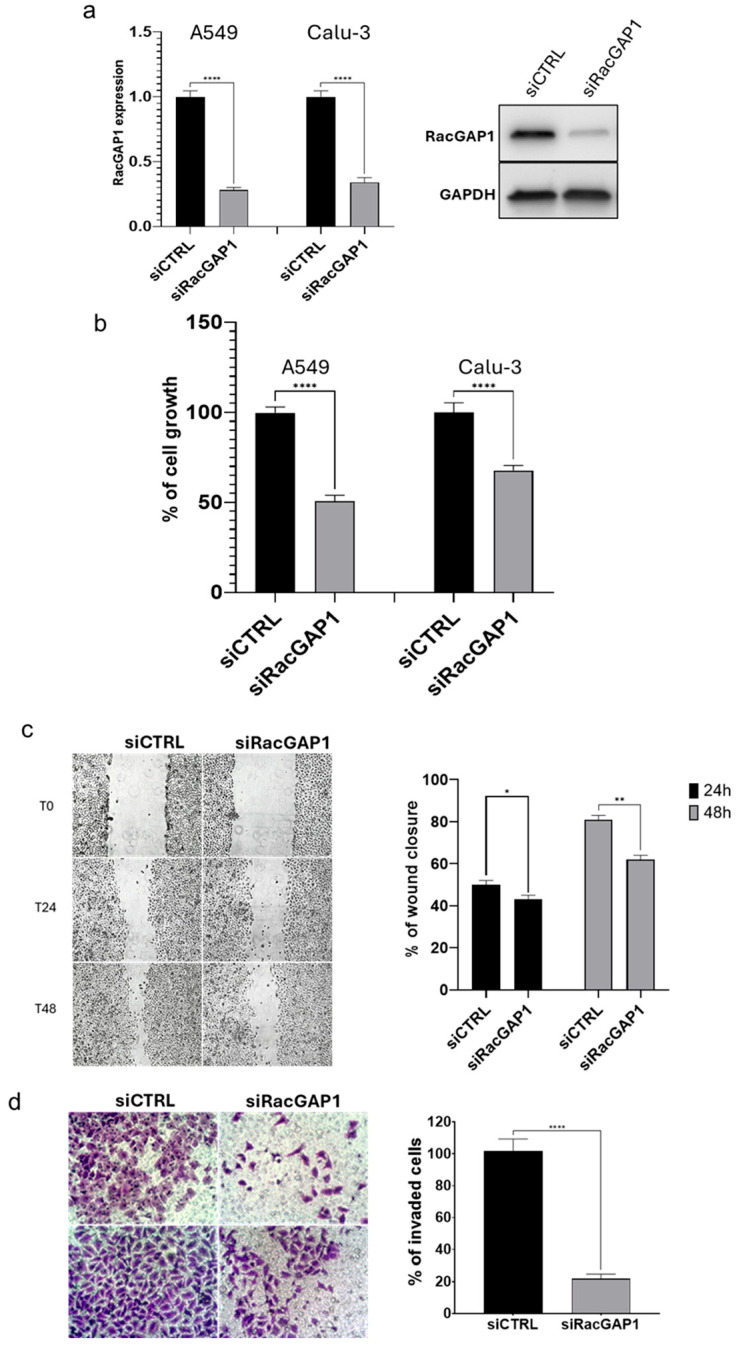
Effect of RacGAP1 silencing on cell proliferation, migration and invasion. Cells were transfected with the indicated molecules, control siRNA molecule (siCTRL) and siRNA against RacGAP1 (siRacGAP1). (**a**) RacGAP1 silencing efficiency was evaluated by determining its expression in the different samples through RT-qPCR (left panel) and Western blot (right panel) after 48 h from transfections. RacGAP1 expression values are reported as fold mean (2^−ΔΔCt^) relative to control (siCTRL) set as 1. (**b**) Cell growth was evaluated by MTT assay 48 h after transfections; the values are reported as percentage from transfection control experiment (siCTRL), which was set to 100. (**c**) Cell migration was assessed by wound-healing assay. Left panel: representative images of migrating cells; Right panel: bar graph showing percentage of wound closure relative to transfection control experiments at 24h and 48h after transfection compared to T0. (**d**) Cell invasion was measured by transwell assay. Left panel: four representative crystal violet-stained fields of invading cells; Right panel: bar graph showing cell counting percentage of invading siRacGAP1 transfected cells relative to siCTRL transfected cells set at 100. Data are the mean ± SD of three independent experiments. * *p* < 0.05, ** *p* < 0.01, **** *p* < 0.0001 for Student’s *t*-test.

Given the observed upregulation of RacGAP1 in LUAD patients and its association with prognostic factors and poor survival, the potential oncogenic role of RacGAP1 in lung adenocarcinoma was experimentally evaluated. Specifically, LUAD cell lines A549 and Calu-3 were transfected with siRNA against RacGAP1 (siRacGAP1) to silence RacGAP1 expression, and the results were compared with those of cells transfected with a siRNA control (siCTRL). First, the effectiveness of RacGAP1 knockdown in LUAD cell lines transfected with siRacGAP1 was evaluated using reverse transcription quantitative PCR (RT-qPCR) and Western blot. As shown in Figure 4a (left panel), siRacGAP1 transfection significantly inhibited RacGAP1 expression by up to 70% (*p* < 0.0001) in both A549 and Calu-3 cell lines compared to siCTRL; consistently, protein level was also strongly reduced by siRacGAP1 transfection (Figure 4a, right panel). The effects of RacGAP1 silencing on cancer hallmarks were then evaluated using cell-based assays, i.e., cell proliferation, migration and invasion. The MTT assay was used to evaluate cell proliferation. As expected for an oncogene, knockdown of RacGAP1 decreased cell proliferation by up to 50% and 35% in A549 and Calu-3, respectively, compared to that of siCTRL-transfected cells (Figure 4b). Then, a wound-healing assay was performed to explore the impact of RacGAP1 on cell migration. The results demonstrated that RacGAP1 silencing significantly inhibited cell migration, reducing the migratory capacity by 20% at 24 h and by 30% at 48 h post-wounding (Figure 4c). Additionally, the effect of RacGAP1 silencing on the invasiveness of A549 cells was assessed via a transwell invasion assay. The results indicate that, compared with the control, RacGAP1 silencing resulted in an 80% reduction in cell invasion (Figure 4d). These results provide evidence that RacGAP1 knockdown impacts proliferation, migration, and invasion in LUAD, indicating its role in promoting lung adenocarcinoma.

### 3.3. RacGAP1 Exerts Its Oncogenic Effect by Regulating the Wnt Pathway

The potential involvement of RacGAP1 in the abnormal activation of the Wnt pathway in LUAD was then investigated based on the following considerations: a plethora of studies showed the pivotal role of the Wnt pathway in cancer, including LC [20]; a potential functional link between RacGAP1 and Wnt pathway has been already suggested by bioinformatics analyses on differentially expressed genes in Aurora kinase A (AURKA)-silenced colorectal cancer cell lines [18]; in addition, expression analyses in gastric cancer patients demonstrated significant correlations between AURKA vs. RacGAP1, and between RacGAP1 vs. β-catenin, at the core of Wnt pathway, thus supporting the functional link, although without mechanistical insights [19]; bioinformatics analysis towards nasopharyngeal carcinoma patients dataset correlated the increased expression of RacGAP1 to Wnt signaling pathway [34].

**Figure 5:**
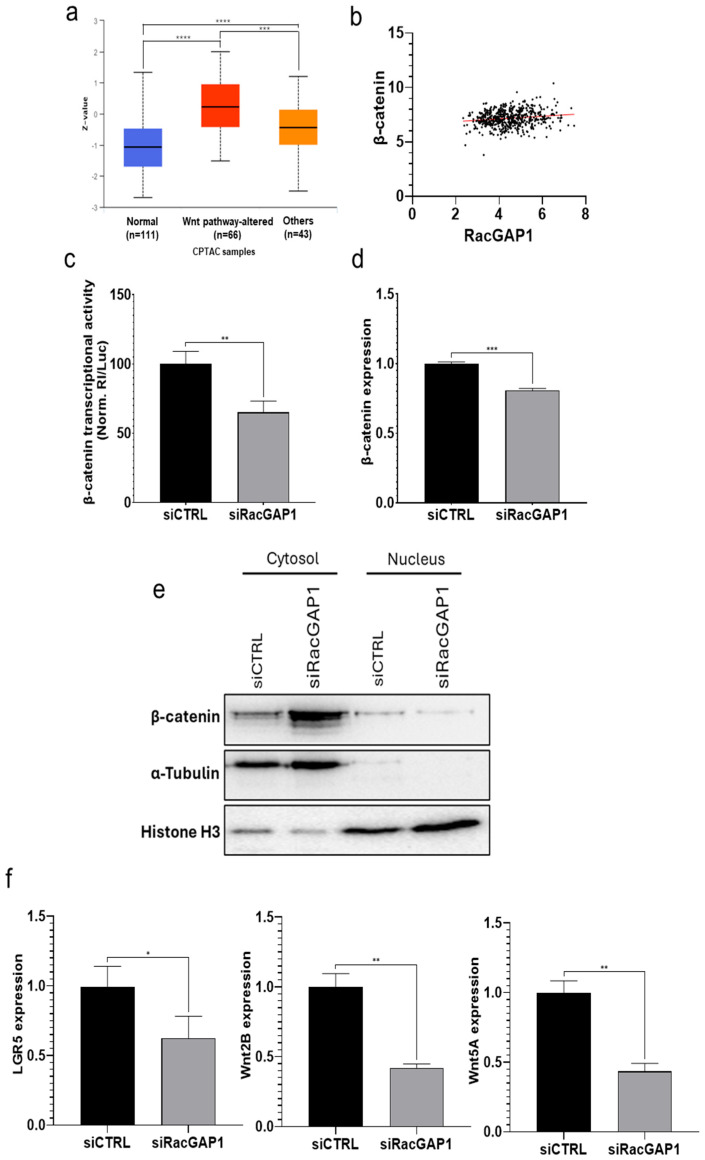
Role of RacGAP1 in the Wnt pathway regulation. (**a**) Box plot reporting RacGAP1 protein levels (Z-value) in normal samples, in patients with altered Wnt pathway status and in patients without Wnt alteration (Others) from Clinical Proteomic Tumor Analysis Consortium (CPTAC) dataset using the UALCAN database. Number of analyzed patients (n =) is reported in parenthesis; (**b**) Scatter plot representing the positive correlation between RacGAP1 and β-catenin expression in the LUAD dataset from TCGA (*r* = 0.164, *p* = 0.0002). (**c**) β-catenin transcriptional activity was evaluated using the TOP flash assay in A549 cells transfected with the indicated molecules. The Luc/Rl ratio (firefly luciferase activity/Renilla reniformis activity) was determined 48 h after transfections and reported as percentage from transfection control experiment (siCTRL), which was set to 100. (**d**) β-catenin expression levels were evaluated by RT-qPCR on RNA purified from A549 48 h after transfections with the indicated molecules. The values are reported as fold mean (2^−ΔΔCt^) relative to control (siCTRL) set as 1. (**e**) Western blot analysis of cytoplasmic and nuclear protein extracts from A549 cells 48 h after transfections with the indicated molecules. Cropped images of blots for β-catenin and loading controls, i.e., α-tubulin and histone H3 for the cytoplasmic and nuclear fractions, respectively. (**f**) Expression levels of Wnt-related genes were evaluated by RT-qPCR on RNA purified from A549 48 h after transfections with the indicated molecules. The values are reported as fold mean (2^−ΔΔCt^) relative to control (siCTRL) set as 1. Data are the mean ± SD of three independent experiments. * *p* < 0.05, ** *p* < 0.01, *** *p* < 0.001, **** *p* < 0.0001 for one-way ANOVA (**a**), Pearson coefficient analysis (**b**) and *t*-test (**c**,**d**,**f**).

First, data from CPTAC via the UALCAN website were analyzed to assess RacGAP1 protein levels in LUAD patients categorized by Wnt pathway status: patients with altered Wnt pathway (“Wnt pathway-altered”), patients without Wnt pathway-altered (“Others”) and healthy patients (“Normal”). The results revealed increased RacGAP1 protein levels in patients with “Wnt pathway-altered” group compared to both the “Others” and “Normal” groups (Figure 5a), indicating the upregulation of RacGAP1 in the context of altered Wnt pathway status. Moreover, the correlation between RacGAP1 expression and the expression of β-catenin, a key player in the Wnt pathway, in the LUAD-TCGA dataset was evaluated using Pearson correlation coefficient analysis. The analysis revealed a positive correlation (*r* = 0.164), weak but statistically significant (*p* = 0.0002) (Figure 5b); these data are particularly meaningful taking into consideration the large number of patients (530 from TCGA dataset). Overall, data from patients suggested that RacGAP1 may influence the Wnt pathway.

Then, to experimentally validate the above hypothesis based on bioinformatics and determine the impact of RacGAP1 on Wnt pathway activation, a TOP flash reporter assay was subsequently performed after RacGAP1 silencing. In brief, the rationale of the assay is based on the fact that if the Wnt pathway is inhibited, luciferase activity driven by the activation of promoter Wnt response elements (WREs) via β-catenin/T-cell factor (TCF)-dependent transcriptional activity is reduced. siRacGAP1 transfection resulted in a reduction of luciferase activity of up to 35% compared with that in siCTRL-transfected cells (Figure 5c), providing evidence for the potential role of RacGAP1 in regulating β-catenin transcriptional activity. Additionally, the results demonstrated that siRacGAP1 transfection significantly reduced β-catenin expression by up to 20% (Figure 5d). To further investigate the role of RacGAP1 in the Wnt pathway, we also analyzed the subcellular localization of β-catenin protein in siRacGAP1-transfected cells. The Western blot results showed that the β-catenin level was increased in the cytoplasmic fraction, whereas it resulted in a strong decrease in the nucleus (Figure 5e), consistently and further supporting the data from the TOP flash reporter assay showing a decreased transcriptional activity. Finally, the impact of RacGAP1 silencing on the expression of downstream Wnt pathway-related genes was assessed, finding that the expression of LGR5 (Leucine Rich Repeat Containing G Protein-Coupled Receptor 5), Wnt2B, and Wnt5A was significantly inhibited, with reductions ranging from 40% to 60% (Figure 5f). These results strongly indicate that RacGAP1 modulates the transcriptional activity of the Wnt/β-catenin pathway, potentially contributing to LUAD progression.

## 4. Discussion

Lung cancer is the most commonly diagnosed cancer and the leading cause of cancer-related death worldwide. NSCLC accounts for approximately 85% of all lung cancer cases, with LUAD emerging as the most prevalent subtype [1,2,4]. RacGAP1, a member of the Rho GAPs family that inactivates Rho GTPases by promoting GTP hydrolysis, is involved in several cellular processes [9,10,11]. Previous studies have demonstrated that aberrant expression of RacGAP1 occurs in different cancer types, including cervical cancer [14], esophageal cancer [15], glioma [16], ovarian cancer [17], colorectal cancer [18], gastric cancer [19] and hepatocellular carcinoma [13]. In HCC, RacGAP1 expression was also identified as a prognostic indicator for early recurrence [35]. With regard to LC, very recently, RacGAP1 was found to be upregulated in tumor samples [28,29,30]; Western blot analyses also indicated the involvement of the PI3K/AKT signaling pathway in the regulation of cell proliferation via RacGAP1 [28]. Importantly, mouse xenograft experiments showed that decreased RacGAP1 expression significantly reduced tumor growth, paralleled by a decreased expression of some pluripotency-related and cell cycle-related genes, thus validating the bioinformatic selection of RacGAP1 as a hub gene of the high stemness score group of LUAD patients and closely correlated with poor prognosis [29]. However, the clinical significance of RacGAP1 overexpression and mechanistic insights on its involvement in LC driving pathways are still largely unknown.

In this study, we found that RacGAP1 was markedly upregulated in five different transcriptomic datasets of patients diagnosed with lung adenocarcinoma (Figure 1a–d). Consistently, our analysis of the UALCAN database revealed that RacGAP1 protein levels were significantly higher in LUAD tissues than in normal lung tissues (Figure 1f). These data demonstrated the upregulation of RacGAP1 at both the mRNA and protein levels in lung adenocarcinoma. Therefore, we investigated the correlation between RacGAP1 overexpression and clinical parameters in LUAD. Our findings revealed that RacGAP1 was upregulated in tumor samples, independently from race and age, in both males and females (Figure 2a–c), and significantly increased along the tumor stage, particularly in stage IV patients (Figure 2d). Furthermore, we found a clear correlation between elevated RacGAP1 expression and unfavorable prognostic factors such as tumor size and lymph node metastasis (Figure 2e,f). Our study also revealed an association between RacGAP1 expression and tobacco smoke, a well-known risk factor for LUAD. Indeed, we found that RacGAP1 expression was significantly higher in smokers than in nonsmokers (Figure 2g). Interestingly, in patients who stopped smoking, the expression level of RacGAP1 was lower than that in smoking patients (Figure 2h). These findings suggest a new potential molecular mechanism for the regulation of RacGAP1 expression related to tobacco smoke. However, other not reported factors, such as air pollution, occupational exposure and genetic background, may influence the association between RacGAP1 expression and smoking habits. Further studies will be required to better assess the association of those factors, RacGAP1 expression and clinicopathological features.

Finally, our in silico analysis revealed that patients with increased RacGAP1 expression had decreased overall and relapse-free survival(Figure 3a–c).

Overall, the results of the described analyses of patient cohorts strongly suggest an oncogenic role of RacGAP1, thus prompting us to experimentally validate its involvement in cancer. In particular, we obtained an effective silencing of RacGAP1 by siRNA transfection with the aim to observe the effect of its absence on cancer hallmarks. Although rescue experiments (e.g., RacGAP1 re-expression after silencing) were not performed, experimental results demonstrated the impact of RacGAP1 on cell proliferation, migration and invasion, thus supporting its oncogenic role in lung adenocarcinoma (Figure 4a–d).

The Wnt/β-catenin signaling pathway is implicated in several cellular processes, such as embryonic development, cell homeostasis and tissue differentiation [36]. Wnt/β-catenin signaling misregulation is emerging as a key player in the onset and progression of human malignancies, including lung cancer. By activating β-catenin, Wnt signaling can control cell proliferation, the cell cycle, migration and invasion [37]. Interestingly, a possible functional link between RacGAP1 and the Wnt/β-catenin signaling pathway has been suggested in nasopharyngeal carcinoma, as well as colorectal and gastric cancers [18,19,34]. Thus, we analyzed RacGAP1 expression in LUAD patients with different Wnt pathway status to investigate the potential involvement of RacGAP1 in the regulation of the Wnt pathway. Our analysis demonstrated that RacGAP1 protein levels were higher in patients with altered Wnt pathway than in patients without Wnt alterations and normal patients (Figure 5a). Additionally, we observed a significant positive correlation between RacGAP1 and β-catenin (Figure 5b), a key factor in the Wnt signaling pathway, in the LUAD-TCGA dataset. Based on the above analyses performed on available patients’ datasets and with the aim to validate and deepen molecular mechanisms functionally linking RacGAP1 to the Wnt/β-catenin signaling pathway, we experimentally demonstrated that RacGAP1 silencing inhibited β-catenin transcriptional activity by using the TOP flash assay (Figure 5c). Given that the stability and nuclear translocation of the intracellular β-catenin are known to impact the Wnt signaling pathway, thereby regulating the expression of different genes involved in cancer onset and progression, the level of β-catenin was also evaluated after RacGAP1 silencing. Our experimental results indicate that RacGAP1 silencing resulted in a slight reduction in β-catenin mRNA expression (Figure 5d); more importantly, its level appeared strongly reduced in the nucleus (Figure 5e). These results indicate that RacGAP1 may modulate the Wnt pathway by regulating β-catenin transcriptional activity and its nuclear level. However, further studies will be required to clarify how RacGAP1 can precisely regulate β-catenin activity and compartmentalization (regulation of degradation and/or nuclear translocation), as well as the contribution of other intermediate molecules potentially involved in the process. Regarding the latter, the involvement of E-cadherin could be investigated, given its ability in binding and sequestering β-catenin from the nuclear signaling pool and experiments reporting that the inhibition of RacGAP1 in HCC cells and xenograft tumors resulted in an enhanced expression of E-cadherin [38,39]. Nevertheless, we moved a step forward by evaluating the expression of Wnt pathway-related genes, finding that LGR5, Wnt2B and Wnt5A were strongly inhibited in RacGAP1-silenced cells (Figure 5f). The Wnt ligands Wnt2B and Wnt5A, secreted glycoproteins interacting with the transmembrane receptors FZD/LRPs (Frizzleds/Low-density lipoprotein receptor-related proteins), are known to play a pivotal role in the regulation of signaling and their aberrant expression has been found to be closely correlated with the occurrence and progression of LC [40]. In particular, with regard to Wnt2B, a recent study demonstrated that, in LC, DDX56 (DEAD-Box Helicase 56) can increase the transcription of the target gene Wnt2B through the degradation of primary miR-378a [41]; additionally, RPPH1 (ribonuclease P RNA component H1), a long non-coding RNA associated with NSCLC progression and cisplatin resistance, is recognized to stimulate the Wnt pathway by elevating Wnt2B expression via the RPPH1/miR-326/Wnt2B axis [42]. With regard to Wnt5A, a more recent study showed that the lncRNA AL139294.1 activated the Wnt and NF-κB2 pathways by interacting with miR-204-5p and upregulating Wnt5A expression [43]. Furthermore, Wnt5A expression was found to be upregulated by 6-pyruvoyl-tetrahydropterin synthase (PTS) [44], circVAPA [45], ATF4 (Activating transcription factor 4) [46] and JPX [47] in LC. LGR5 has been identified as a stem cell-specific receptor to promote the canonical Wnt/β-catenin signaling pathway. In the presence of R-spondin (RSPO), the N-terminal domain of LGR5 binds to RSPO and interacts with Wnt ligands, supporting Wnt/β-catenin signaling [48]. Overall, our results indicated that RacGAP1 silencing resulted in lower levels of β-catenin in the nucleus, inhibition of its transcriptional activity, and the downregulation of the Wnt-related genes LGR5, Wnt2B and Wnt5A.

## 5. Conclusions

Multiple analyses towards different patients’ datasets revealed that RacGAP1 is upregulated in LUAD and is correlated with unfavorable prognostic factors and poor survival. Then, experimental analyses validated the oncogenic role of RacGAP1, since RacGAP1 knockdown was observed to inhibit cell proliferation, migration and invasion. Finally, we investigated the potential molecular mechanism by which RacGAP1 regulates the Wnt pathway.

Overall, the results suggest that the increased expression of RacGAP1 in tumors may modulate the activity of the Wnt pathway by promoting β-catenin transcriptional activity and elevating Wnt5a, Wnt5b and LGR5 expression, thus enhancing the pathway. Therefore, in this study, we demonstrated for the first time that RacGAP1 plays a pivotal role in lung adenocarcinoma, contributing to the abnormal activation of the Wnt/β-catenin signaling pathway. These findings may provide new opportunities for innovative therapeutic strategies and the development of advanced diagnostic panels.

## Data Availability

All data supporting the findings of this study are available within the paper. The anonymized data collected are available as open data via R2 (Genomics analysis and visualization platform, https://r2.amc.nl accessed on 10 October 2023) and UALCAN (The University of ALabama at Birmingham CANcer data analysis Portal, https://ualcan.path.uab.edu/index.html accessed on 10 October 2023).

## Abbreviations

The following abbreviations are used in this manuscript:

RacGAP1	Rac GTPase activating protein 1
LC	lung cancer
SCLC	small cell lung cancer
NSCLC	non-small cell lung cancer
LUAD	lung adenocarcinoma
LUSC	lung squamous cell carcinoma
Rho GAPs	Rho GTPase-activating proteins
HCC	hepatocellular carcinoma
WRE	Wnt response element
TCF	T-cell factor
LRG5	Leucine Rich Repeat Containing G Protein-Coupled Receptor 5
FZD/LRP5	Frizzleds/Low-density lipoprotein receptor-related protein
RPPH1	ribonuclease P RNA component H1
DDX56	DEAD-Box Helicase 56
PST	6-pyruvoyl-tetrahydropterin synthase
ATF4	Activating transcription factor 4
RSPO	R-spondin
